# Supplementary material for: Pathophysiological Significance of Neutrophilic Transfer RNA-Derived Small RNAs in Asymptomatic Moyamoya Disease
Source: Cells. 2021 May 1;10(5):1086. doi: 10.3390/cells10051086 (PMC8147334; doi:10.3390/cells10051086)

2D Structure	Local AU	Position

```
1467                          8mer-1a              1480
5 ' - a g t G A G T C A C A G G a - 3 ' utr
                :  |  |  |  |  |  |  |  |  |
3 ' - g u u U U C A G U G U C C g - 5 ' tRF
16 15     14 13         7  6  5  4  3  2
  3' Pairing               Seed
```

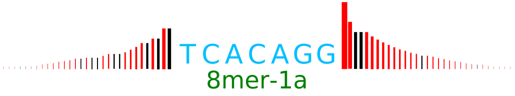

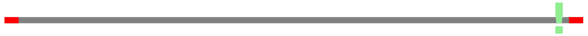

Supplement: Supplementary file 1 [file cells-10-01086-s001.zip › cells-1149161 3rd supplementary materials/Data Supplements/Data Supplement V.zip!/Data supplement 6-mRNAs targets of tsRNAs/tsRNA-Arg-CCT-008/Diagrams/tRF-Arg-CCT-008_vs_ENST00000343949(ZNF763).pdf]
